# Raltegravir monohydrate

**DOI:** 10.1107/S1600536813029747

**Published:** 2013-11-06

**Authors:** Thammarse S. Yamuna, Jerry P. Jasinski, Brian J. Anderson, H. S. Yathirajan, Manpreet Kaur

**Affiliations:** a Department of Studies in Chemistry, University of Mysore, Manasagangotri, Mysore 570 006, India; b Department of Chemistry, Keene State College, 229 Main Street, Keene, NH 03435-2001, USA

## Abstract

The hydrated title compound [systematic name: *N*-(4-fluoro­benz­yl)-5-hy­droxy-1-methyl-2-{1-methyl-1-[(5-methyl-1,3,4-oxa­diazol-2-ylcarbon­yl)amino]­eth­yl}-6-oxo-1,6-di­hydro­pyrimidine-4-carb­oxamide monohydrate], C_20_H_21_FN_6_O_5_·H_2_O, is recognised as the first HIV integrase inhibitor. In the mol­ecule, the dihedral angles between the mean planes of the pyrimidine ring and the phenyl and oxa­diazole rings are 72.0 (1) and 61.8 (3)°, respectively. The mean plane of the oxa­diazole ring is twisted by 15.6 (3)° from that of the benzene ring, while the mean plane of amide group bound to the oxadiaole ring is twisted by 18.8 (3)° from its mean plane. Intra­molecular O—H⋯O and C—H⋯N hydrogen bonds are observed in the mol­ecule. The crystal packing features O—H⋯O hydrogen bonds, which include bifurcated O—H⋯(O,O) hydrogen bonds from one H atom of the water mol­ecule. In addition, N—H⋯O hydrogen bonds are observed involving the two amide groups. These inter­actions link the mol­ecules into chains along [010].

## Related literature

For general background to and pharmacological properties of Raltegravir, see: Burger (2010 ▶); Cocohoba & Dong (2008 ▶); Croxtall & Keam (2009 ▶); Evering & Markowitz (2008 ▶); Hicks & Gulick (2009 ▶); Savarino (2006 ▶); Temesgen & Siraj (2008 ▶). For related structures, see: Fun *et al.* (2011 ▶); Shang *et al.* (2012 ▶); Shang, Ha *et al.* (2011 ▶); Shang, Qi *et al.* (2011 ▶); Thiruvalluvar *et al.* (2007 ▶). For standard bond lengths, see: Allen *et al.* (1987 ▶).
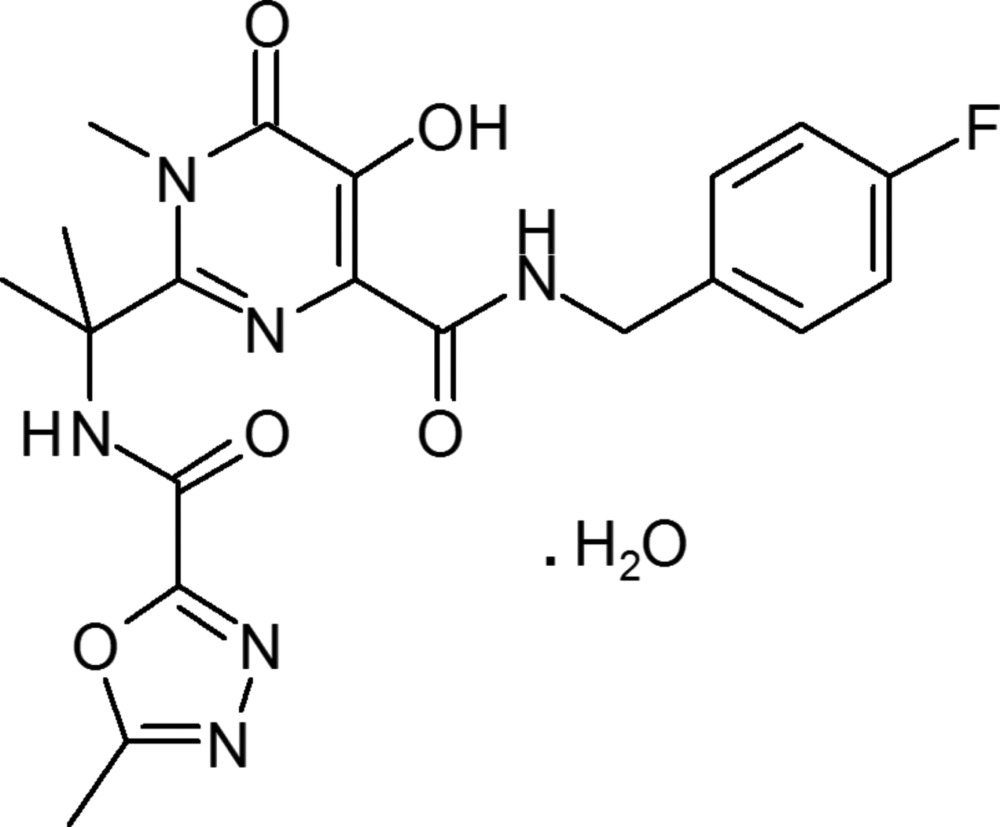


## Experimental

### 

#### Crystal data

C_20_H_21_FN_6_O_5_·H_2_O
*M* *_r_* = 462.44
Triclinic, 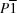

*a* = 8.3860 (6) Å
*b* = 11.8610 (9) Å
*c* = 12.1102 (9) Å
α = 110.481 (7)°
β = 108.093 (7)°
γ = 92.329 (6)°
*V* = 1057.44 (15) Å^3^
*Z* = 2
Mo *K*α radiation
μ = 0.12 mm^−1^
*T* = 173 K
0.44 × 0.32 × 0.26 mm

#### Data collection

Agilent Xcalibur (Eos, Gemini) diffractometer
Absorption correction: multi-scan (*CrysAlis PRO* and *CrysAlis RED*; Agilent, 2012 ▶) *T*_min_ = 0.890, *T*_max_ = 1.000
12734 measured reflections
7007 independent reflections
5042 reflections with *I* > 2σ(*I*)
*R*_int_ = 0.025

#### Refinement

*R*[*F* ^2^ > 2σ(*F* ^2^)] = 0.067
*wR*(*F* ^2^) = 0.206
*S* = 1.03
7007 reflections
306 parameters
H-atom parameters constrained
Δρ_max_ = 0.78 e Å^−3^
Δρ_min_ = −0.39 e Å^−3^

### 

Data collection: *CrysAlis PRO* (Agilent, 2012 ▶); cell refinement: *CrysAlis PRO*; data reduction: *CrysAlis RED* (Agilent, 2012 ▶); program(s) used to solve structure: *SUPERFLIP* (Palatinus & Chapuis, 2007 ▶); program(s) used to refine structure: *SHELXL2012* (Sheldrick, 2008 ▶); molecular graphics: *OLEX2* (Dolomanov *et al.*, 2009 ▶); software used to prepare material for publication: *OLEX2*.

## Supplementary Material

Crystal structure: contains datablock(s) I. DOI: 10.1107/S1600536813029747/sj5363sup1.cif

Structure factors: contains datablock(s) I. DOI: 10.1107/S1600536813029747/sj5363Isup2.hkl

Supplementary material file. DOI: 10.1107/S1600536813029747/sj5363Isup3.cml

Additional supplementary materials:  crystallographic information; 3D view; checkCIF report

## Figures and Tables

**Table 1:** Hydrogen-bond geometry (Å, °)

*D*—H⋯*A*	*D*—H	H⋯*A*	*D*⋯*A*	*D*—H⋯*A*
O4—H4⋯O5	0.84	1.88	2.593 (2)	143
C20—H20*C*⋯N1	0.98	2.25	2.982 (3)	130
N1—H1⋯O5^i^	0.88	2.23	2.970 (2)	142
N4—H4*A*⋯O1*W* ^ii^	0.88	2.48	3.074 (3)	126
C7—H7*A*⋯O1*W* ^ii^	0.99	2.58	3.240 (3)	124
C10—H10⋯O5^iii^	0.95	2.50	3.393 (3)	158
C20—H20*A*⋯O4^iv^	0.98	2.46	3.394 (2)	160
C20—H20*B*⋯N6^v^	0.98	2.50	3.422 (3)	158
O1*W*—H1*WA*⋯O4^vi^	0.85	2.51	3.249 (3)	146
O1*W*—H1*WA*⋯O3^vi^	0.85	2.33	3.013 (3)	138
O1*W*—H1*WB*⋯O2	0.85	2.04	2.869 (2)	164

Symmetry codes: (i) 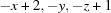; (ii) 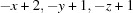; (iii) 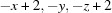; (iv) 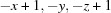; (v) 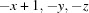; (vi) 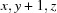.

**Fig. 1.**
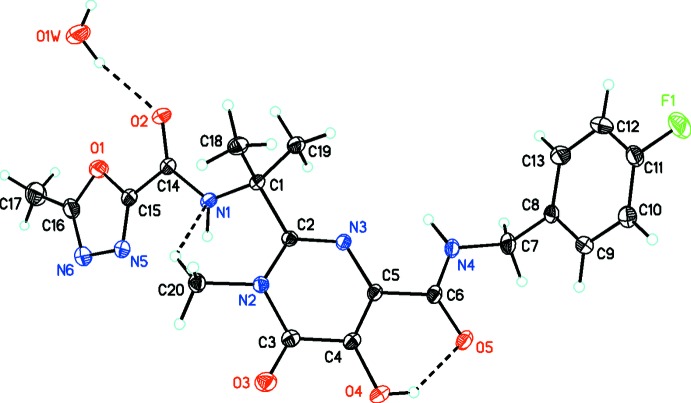
ORTEP drawing of (I) (C20H21FN6O5. H2O) showing the labeling scheme with 30% probability displacement ellipsoids. Dashed lines indicate intramolecular O4—H4···O5, C20—H20C···N1 and intermolecular O1W—H1WB···O2 hydrogen bonds in the asymmentric unit.

**Fig. 2.**
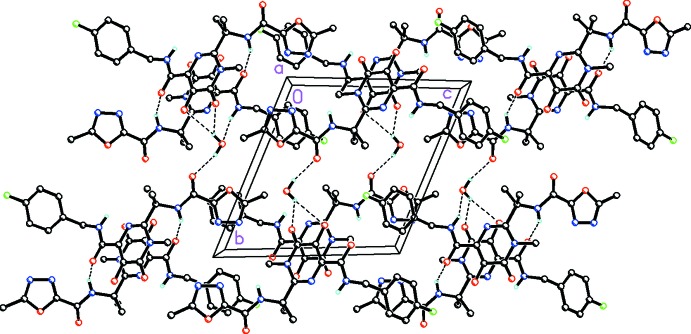
Molecular packing for (I) viewed along the a axis. Dashed lines indicate intermolecular N—H···O and O—H···O hydrogen bonds. H atoms not involved in hydrogen bonding have been removed for clarity.

## supplementary crystallographic information

### 1. Comment

Raltegravir (systematic name:5-hydroxy-1-methyl-2-{1-methyl-1-[(5-methyl-[1,3,4]oxadiazole-2-carbonyl)amino]ethyl}-6-oxo-1,6-dihydropyrimidine-4-carboxylic acid 4-fluorobenzylamide) monohydrate is the first in a novel class of HIV-1 integrase strand-transfer inhibitors with potent antiretroviral activity (Savarino, 2006; Hicks & Gulick, 2009; Evering & Markowitz, 2008; Temesgen & Siraj, 2008). It inhibits the action of the HIV-1-specific enzyme that is responsible for the insertion of viral complimentary DNA into the host genome (Croxtall & Keam, 2009). It is also found to be a generally well tolerated antiretroviral agent that may play an important role in the treatment of patients harboring resistance to other antiretroviral drugs (Cocohoba & Dong, 2008). A review of the pharmacokinetics, pharmacology and clinical studies of Raltegravir has been published (Burger, 2010). The crystal structures of some related compounds, viz., 5-[(4,6-dimethylpyrimidin-2- ylsulfanyl)methyl]-3-(morpholinomethyl)-1,3,4-oxadiazole-2(3H)-thione (Thiruvalluvar *et al.*, 2007), methyl 2-[2-(benzyloxycarbonylamino)propan-2-yl]-5-hydroxy-6-methoxypyrimidine- 4-carboxylate (Fun *et al.*, 2011), methyl 2-(2-{[(benzyloxy)carbonyl]- amino}propan-2-yl)-5-hydroxy-6-oxo-1,6-dihydropyrimidine-4-carboxylate (Shang, Ha *et al.*, 2011), methyl 2-(2-{[(benzyloxy)carbonyl]amino}propan-2-yl)- 5-hydroxy-6-methoxypyrimidine-4-carboxylate (Shang, Qi *et al.*, 2011) and methyl 2-[2-(benzyloxycarbonylamino)propan-2-yl]-5-hydroxy-1-methyl-6-oxo- 1,6-dihydro pyrimidine-4-carboxylate (Shang *et al.*, 2012) have been reported. In view of the importance of Raltegravir, this paper reports the crystal structure of (I), C_20_H_21_FN_6_O_5_. H_2_O.

In the title compound, (I), the dihedral angles between the mean planes of the pyrimidine ring and the phenyl and oxadiazole rings are 72.0 (1)° and 61.8 (3)° respectively (Fig. 1). The mean plane of the oxadiazole ring is twisted by 15.63° from that of the phenyl ring. In addition, the mean plane of the N1–C14–O2 amide group adjacent to the oxadiazole ring is twisted by 18.8 (3)° from the mean plane of the oxidiazole ring. Bond lengths are within normal ranges (Allen *et al.*, 1987). Intramolecular O—H···O and C—H···N hydrogen bonds are observed in the molecule (Table. 1).

The crystal packing is stabilized by intermolecular O—H···O hydrogen bonds which include bifurcated O1W–H1WA···O3 and O1W–H1WA···O4 hydrogen bonds from the H1WA atom of the water molecule. In addition, intermolecular N1–H1···O5 and N4–H4A···O1W hydrogen bonds involving the two amide groups are also observed. These interactions link the molecules into chains along [0 1 0].

### 2. Experimental

Raltegravir (CAS No. 518048-05-0) (0.2 g) was dissolved in a 1:1:1(v/v) mixture of methanol, dimethyl sulfoxide and dimethyl formamide at 308 K and left for slow evaporation. Crystals suitable for X-ray work were obtained after a few months (m.p.: 383–388 K).

### 3. Refinement

H1WA and H1WB were located in a difference map and refined isotropically. All other H atoms were placed in their calculated positions and then refined using a riding model with Atom—H lengths of 0.95Å (CH), 0.99Å (CH_2_), 0.98Å (CH_3_), 0.88Å (NH) or 0.84Å (OH). Isotropic displacement parameters for these atoms were set to 1.2 (CH, CH_2_, NH) or 1.5 (CH_3_, OH, OH_2_) times *U*_eq_ of the parent atom. Idealised Me and tetrahedral OH (O4(H4))were refined as rotating groups. The highest peak (-0.783) in the final difference map is located 1.02 Å from O1.

### Crystal data

**Table:**

C_20_H_21_FN_6_O_5_·H_2_O	*Z* = 2
*M**_r_* = 462.44	*F*(000) = 484
Triclinic, *P*1	*D*_x_ = 1.452 Mg m^−^^3^
*a* = 8.3860 (6) Å	Mo *K*α radiation, λ = 0.71073 Å
*b* = 11.8610 (9) Å	Cell parameters from 3215 reflections
*c* = 12.1102 (9) Å	θ = 3.0–32.9°
α = 110.481 (7)°	µ = 0.12 mm^−^^1^
β = 108.093 (7)°	*T* = 173 K
γ = 92.329 (6)°	Irregular, colourless
*V* = 1057.44 (15) Å^3^	0.44 × 0.32 × 0.26 mm

### Data collection

**Table:**

Agilent Xcalibur (Eos, Gemini) diffractometer	7007 independent reflections
Radiation source: Enhance (Mo) X-ray Source	5042 reflections with *I* > 2σ(*I*)
Detector resolution: 16.0416 pixels mm^-1^	*R*_int_ = 0.025
ω scans	θ_max_ = 33.0°, θ_min_ = 3.1°
Absorption correction: multi-scan (*CrysAlis PRO* and *CrysAlis RED*; Agilent, 2012)	*h* = −12→12
*T*_min_ = 0.890, *T*_max_ = 1.000	*k* = −14→17
12734 measured reflections	*l* = −17→18

### Refinement

**Table:**

Refinement on *F*^2^	Primary atom site location: structure-invariant direct methods
Least-squares matrix: full	Hydrogen site location: mixed
*R*[*F*^2^ > 2σ(*F*^2^)] = 0.067	H-atom parameters constrained
*wR*(*F*^2^) = 0.206	*w* = 1/[σ^2^(*F*_o_^2^) + (0.0974*P*)^2^ + 0.6433*P*] where *P* = (*F*_o_^2^ + 2*F*_c_^2^)/3
*S* = 1.03	(Δ/σ)_max_ < 0.001
7007 reflections	Δρ_max_ = 0.78 e Å^−^^3^
306 parameters	Δρ_min_ = −0.39 e Å^−^^3^
0 restraints	

### Special details

**Table:**

Geometry. All esds (except the esd in the dihedral angle between two l.s. planes) are estimated using the full covariance matrix. The cell esds are taken into account individually in the estimation of esds in distances, angles and torsion angles; correlations between esds in cell parameters are only used when they are defined by crystal symmetry. An approximate (isotropic) treatment of cell esds is used for estimating esds involving l.s. planes.

### Fractional atomic coordinates and isotropic or equivalent isotropic displacement parameters (Å^2^)

**Table:**

	*x*	*y*	*z*	*U*_iso_*/*U*_eq_	
F1	1.1554 (2)	0.33688 (15)	1.31637 (13)	0.0578 (4)	
O1	0.7785 (2)	0.36533 (14)	0.09901 (14)	0.0412 (4)	
O2	0.7121 (2)	0.44719 (13)	0.32679 (14)	0.0398 (4)	
O3	0.50567 (19)	−0.13326 (13)	0.34626 (15)	0.0355 (3)	
O4	0.76855 (19)	−0.16476 (12)	0.52110 (14)	0.0320 (3)	
H4	0.8570	−0.1629	0.5789	0.048*	
O5	1.04993 (19)	−0.05743 (13)	0.70762 (13)	0.0315 (3)	
N1	0.7540 (2)	0.26143 (14)	0.33868 (14)	0.0273 (3)	
H1	0.7661	0.1875	0.2950	0.033*	
N2	0.62443 (18)	0.06543 (13)	0.40189 (13)	0.0221 (3)	
N3	0.87875 (19)	0.15547 (13)	0.57317 (13)	0.0228 (3)	
N4	1.1487 (2)	0.14234 (15)	0.76091 (14)	0.0282 (3)	
H4A	1.1334	0.2069	0.7414	0.034*	
N5	0.7269 (2)	0.17737 (16)	0.08576 (16)	0.0347 (4)	
N6	0.7475 (3)	0.17321 (18)	−0.02828 (18)	0.0427 (5)	
C1	0.7546 (2)	0.28635 (15)	0.46715 (16)	0.0238 (3)	
C2	0.7531 (2)	0.16307 (15)	0.48264 (15)	0.0211 (3)	
C3	0.6221 (2)	−0.04688 (16)	0.41474 (17)	0.0245 (3)	
C4	0.7677 (2)	−0.05480 (15)	0.51363 (16)	0.0237 (3)	
C5	0.8866 (2)	0.04642 (15)	0.58924 (15)	0.0222 (3)	
C6	1.0360 (2)	0.04108 (16)	0.69173 (16)	0.0247 (3)	
C7	1.2983 (3)	0.1521 (2)	0.86874 (18)	0.0331 (4)	
H7A	1.3951	0.2062	0.8731	0.040*	
H7B	1.3306	0.0705	0.8568	0.040*	
C8	1.2642 (2)	0.20230 (17)	0.99030 (17)	0.0271 (4)	
C9	1.1883 (3)	0.12534 (17)	1.02977 (18)	0.0304 (4)	
H9	1.1619	0.0401	0.9810	0.036*	
C10	1.1498 (3)	0.17008 (19)	1.13923 (19)	0.0333 (4)	
H10	1.0959	0.1170	1.1652	0.040*	
C11	1.1919 (3)	0.2928 (2)	1.20832 (18)	0.0370 (5)	
C12	1.2710 (4)	0.3723 (2)	1.1741 (2)	0.0527 (7)	
H12	1.3007	0.4570	1.2252	0.063*	
C13	1.3067 (4)	0.3264 (2)	1.0637 (2)	0.0438 (5)	
H13	1.3604	0.3800	1.0383	0.053*	
C14	0.7364 (3)	0.34282 (17)	0.28320 (18)	0.0297 (4)	
C15	0.7465 (3)	0.28953 (17)	0.15403 (18)	0.0312 (4)	
C16	0.7766 (3)	0.2857 (2)	−0.0153 (2)	0.0388 (5)	
C17	0.8092 (5)	0.3338 (3)	−0.1049 (3)	0.0595 (7)	
H17A	0.7148	0.3741	−0.1349	0.089*	
H17B	0.8190	0.2664	−0.1763	0.089*	
H17C	0.9153	0.3928	−0.0628	0.089*	
C18	0.6015 (3)	0.34431 (18)	0.4921 (2)	0.0330 (4)	
H18A	0.6208	0.4311	0.5063	0.049*	
H18B	0.5885	0.3358	0.5666	0.049*	
H18C	0.4979	0.3029	0.4192	0.049*	
C19	0.9184 (3)	0.37382 (17)	0.56034 (19)	0.0319 (4)	
H19A	1.0168	0.3375	0.5464	0.048*	
H19B	0.9234	0.3889	0.6464	0.048*	
H19C	0.9198	0.4512	0.5480	0.048*	
C20	0.4769 (2)	0.06994 (17)	0.29878 (17)	0.0276 (4)	
H20A	0.3837	0.0935	0.3300	0.041*	
H20B	0.4396	−0.0107	0.2302	0.041*	
H20C	0.5098	0.1300	0.2677	0.041*	
O1W	0.6377 (4)	0.63744 (18)	0.2332 (2)	0.0697 (7)	
H1WA	0.6255	0.6900	0.2977	0.105*	
H1WB	0.6630	0.5741	0.2478	0.105*	

### Atomic displacement parameters (Å^2^)

**Table:**

	*U*^11^	*U*^22^	*U*^33^	*U*^12^	*U*^13^	*U*^23^
F1	0.0781 (11)	0.0643 (10)	0.0310 (7)	0.0282 (8)	0.0259 (7)	0.0103 (7)
O1	0.0611 (10)	0.0302 (7)	0.0326 (7)	0.0038 (7)	0.0138 (7)	0.0148 (6)
O2	0.0591 (10)	0.0230 (7)	0.0362 (8)	0.0116 (6)	0.0114 (7)	0.0141 (6)
O3	0.0303 (7)	0.0291 (7)	0.0434 (8)	0.0000 (5)	0.0075 (6)	0.0146 (6)
O4	0.0379 (7)	0.0250 (6)	0.0390 (8)	0.0094 (5)	0.0142 (6)	0.0177 (6)
O5	0.0391 (7)	0.0331 (7)	0.0319 (7)	0.0168 (6)	0.0152 (6)	0.0199 (6)
N1	0.0373 (8)	0.0221 (7)	0.0258 (7)	0.0095 (6)	0.0117 (6)	0.0117 (6)
N2	0.0222 (6)	0.0234 (7)	0.0214 (6)	0.0066 (5)	0.0083 (5)	0.0087 (5)
N3	0.0263 (7)	0.0235 (7)	0.0208 (6)	0.0081 (5)	0.0089 (5)	0.0099 (5)
N4	0.0308 (8)	0.0335 (8)	0.0224 (7)	0.0104 (6)	0.0076 (6)	0.0139 (6)
N5	0.0431 (10)	0.0295 (8)	0.0276 (8)	0.0060 (7)	0.0050 (7)	0.0127 (6)
N6	0.0521 (11)	0.0388 (10)	0.0306 (9)	0.0076 (8)	0.0060 (8)	0.0128 (7)
C1	0.0277 (8)	0.0210 (7)	0.0231 (7)	0.0080 (6)	0.0076 (6)	0.0096 (6)
C2	0.0237 (7)	0.0206 (7)	0.0209 (7)	0.0070 (6)	0.0100 (6)	0.0078 (6)
C3	0.0261 (8)	0.0239 (8)	0.0275 (8)	0.0064 (6)	0.0133 (7)	0.0105 (6)
C4	0.0284 (8)	0.0235 (8)	0.0264 (8)	0.0099 (6)	0.0154 (7)	0.0123 (6)
C5	0.0267 (8)	0.0247 (8)	0.0201 (7)	0.0104 (6)	0.0115 (6)	0.0109 (6)
C6	0.0308 (8)	0.0305 (8)	0.0210 (7)	0.0140 (7)	0.0146 (7)	0.0133 (6)
C7	0.0287 (9)	0.0455 (11)	0.0261 (8)	0.0126 (8)	0.0067 (7)	0.0166 (8)
C8	0.0263 (8)	0.0295 (9)	0.0225 (8)	0.0076 (7)	0.0030 (6)	0.0109 (7)
C9	0.0356 (9)	0.0258 (8)	0.0255 (8)	0.0037 (7)	0.0072 (7)	0.0078 (7)
C10	0.0339 (10)	0.0377 (10)	0.0282 (9)	0.0043 (8)	0.0091 (8)	0.0139 (8)
C11	0.0439 (11)	0.0408 (11)	0.0233 (8)	0.0162 (9)	0.0095 (8)	0.0095 (8)
C12	0.086 (2)	0.0259 (10)	0.0368 (12)	0.0083 (11)	0.0186 (12)	0.0033 (9)
C13	0.0619 (15)	0.0297 (10)	0.0376 (11)	−0.0005 (10)	0.0146 (10)	0.0137 (9)
C14	0.0366 (9)	0.0243 (8)	0.0270 (8)	0.0046 (7)	0.0059 (7)	0.0129 (7)
C15	0.0365 (10)	0.0267 (9)	0.0292 (9)	0.0036 (7)	0.0054 (7)	0.0146 (7)
C16	0.0408 (11)	0.0407 (11)	0.0289 (9)	0.0022 (9)	0.0056 (8)	0.0126 (8)
C17	0.080 (2)	0.0575 (16)	0.0426 (13)	0.0024 (14)	0.0169 (13)	0.0253 (12)
C18	0.0334 (9)	0.0293 (9)	0.0374 (10)	0.0141 (7)	0.0147 (8)	0.0109 (8)
C19	0.0322 (9)	0.0241 (8)	0.0343 (9)	0.0027 (7)	0.0041 (8)	0.0118 (7)
C20	0.0241 (8)	0.0307 (9)	0.0265 (8)	0.0061 (7)	0.0060 (7)	0.0113 (7)
O1W	0.121 (2)	0.0388 (10)	0.0519 (11)	0.0309 (12)	0.0242 (13)	0.0241 (9)

### Geometric parameters (Å, º)

**Table:**

F1—C11	1.364 (2)	C7—H7B	0.9900
O1—C15	1.355 (2)	C7—C8	1.505 (3)
O1—C16	1.371 (3)	C8—C9	1.384 (3)
O2—C14	1.217 (2)	C8—C13	1.389 (3)
O3—C3	1.229 (2)	C9—H9	0.9500
O4—H4	0.8400	C9—C10	1.389 (3)
O4—C4	1.339 (2)	C10—H10	0.9500
O5—C6	1.253 (2)	C10—C11	1.366 (3)
N1—H1	0.8800	C11—C12	1.376 (4)
N1—C1	1.477 (2)	C12—H12	0.9500
N1—C14	1.345 (2)	C12—C13	1.388 (4)
N2—C2	1.383 (2)	C13—H13	0.9500
N2—C3	1.394 (2)	C14—C15	1.500 (3)
N2—C20	1.478 (2)	C16—C17	1.476 (4)
N3—C2	1.296 (2)	C17—H17A	0.9800
N3—C5	1.375 (2)	C17—H17B	0.9800
N4—H4A	0.8800	C17—H17C	0.9800
N4—C6	1.323 (3)	C18—H18A	0.9800
N4—C7	1.469 (2)	C18—H18B	0.9800
N5—N6	1.429 (3)	C18—H18C	0.9800
N5—C15	1.268 (3)	C19—H19A	0.9800
N6—C16	1.291 (3)	C19—H19B	0.9800
C1—C2	1.538 (2)	C19—H19C	0.9800
C1—C18	1.542 (3)	C20—H20A	0.9800
C1—C19	1.529 (3)	C20—H20B	0.9800
C3—C4	1.454 (2)	C20—H20C	0.9800
C4—C5	1.357 (3)	O1W—H1WA	0.8500
C5—C6	1.487 (2)	O1W—H1WB	0.8504
C7—H7A	0.9900		
			
C15—O1—C16	102.68 (16)	C9—C10—H10	121.1
C4—O4—H4	109.5	C11—C10—C9	117.9 (2)
C1—N1—H1	117.4	C11—C10—H10	121.1
C14—N1—H1	117.4	F1—C11—C10	118.0 (2)
C14—N1—C1	125.12 (15)	F1—C11—C12	119.3 (2)
C2—N2—C3	121.45 (14)	C10—C11—C12	122.7 (2)
C2—N2—C20	124.45 (14)	C11—C12—H12	120.6
C3—N2—C20	114.08 (14)	C11—C12—C13	118.7 (2)
C2—N3—C5	119.27 (15)	C13—C12—H12	120.6
C6—N4—H4A	118.4	C8—C13—H13	119.9
C6—N4—C7	123.12 (16)	C12—C13—C8	120.2 (2)
C7—N4—H4A	118.4	C12—C13—H13	119.9
C15—N5—N6	106.18 (17)	O2—C14—N1	126.78 (19)
C16—N6—N5	105.55 (18)	O2—C14—C15	121.48 (17)
N1—C1—C2	106.42 (13)	N1—C14—C15	111.74 (16)
N1—C1—C18	113.17 (14)	O1—C15—C14	119.28 (17)
N1—C1—C19	108.21 (15)	N5—C15—O1	113.46 (18)
C2—C1—C18	110.32 (15)	N5—C15—C14	127.26 (17)
C19—C1—C2	110.05 (14)	O1—C16—C17	119.6 (2)
C19—C1—C18	108.63 (15)	N6—C16—O1	112.1 (2)
N2—C2—C1	120.93 (14)	N6—C16—C17	128.3 (2)
N3—C2—N2	122.32 (15)	C16—C17—H17A	109.5
N3—C2—C1	116.75 (15)	C16—C17—H17B	109.5
O3—C3—N2	122.18 (16)	C16—C17—H17C	109.5
O3—C3—C4	122.56 (16)	H17A—C17—H17B	109.5
N2—C3—C4	115.25 (15)	H17A—C17—H17C	109.5
O4—C4—C3	114.82 (16)	H17B—C17—H17C	109.5
O4—C4—C5	126.23 (16)	C1—C18—H18A	109.5
C5—C4—C3	118.95 (15)	C1—C18—H18B	109.5
N3—C5—C6	117.28 (15)	C1—C18—H18C	109.5
C4—C5—N3	122.65 (15)	H18A—C18—H18B	109.5
C4—C5—C6	120.04 (15)	H18A—C18—H18C	109.5
O5—C6—N4	123.62 (16)	H18B—C18—H18C	109.5
O5—C6—C5	119.27 (17)	C1—C19—H19A	109.5
N4—C6—C5	117.11 (15)	C1—C19—H19B	109.5
N4—C7—H7A	109.3	C1—C19—H19C	109.5
N4—C7—H7B	109.3	H19A—C19—H19B	109.5
N4—C7—C8	111.60 (16)	H19A—C19—H19C	109.5
H7A—C7—H7B	108.0	H19B—C19—H19C	109.5
C8—C7—H7A	109.3	N2—C20—H20A	109.5
C8—C7—H7B	109.3	N2—C20—H20B	109.5
C9—C8—C7	120.34 (17)	N2—C20—H20C	109.5
C9—C8—C13	119.05 (19)	H20A—C20—H20B	109.5
C13—C8—C7	120.60 (19)	H20A—C20—H20C	109.5
C8—C9—H9	119.3	H20B—C20—H20C	109.5
C8—C9—C10	121.39 (18)	H1WA—O1W—H1WB	109.4
C10—C9—H9	119.3		
			
F1—C11—C12—C13	−179.7 (2)	C4—C5—C6—N4	178.97 (16)
O2—C14—C15—O1	19.2 (3)	C5—N3—C2—N2	0.9 (2)
O2—C14—C15—N5	−160.2 (2)	C5—N3—C2—C1	−178.19 (14)
O3—C3—C4—O4	2.5 (3)	C6—N4—C7—C8	−94.3 (2)
O3—C3—C4—C5	−176.74 (17)	C7—N4—C6—O5	−3.5 (3)
O4—C4—C5—N3	178.19 (16)	C7—N4—C6—C5	177.18 (16)
O4—C4—C5—C6	0.3 (3)	C7—C8—C9—C10	−177.60 (18)
N1—C1—C2—N2	−57.7 (2)	C7—C8—C13—C12	178.4 (2)
N1—C1—C2—N3	121.37 (16)	C8—C9—C10—C11	−1.1 (3)
N1—C14—C15—O1	−161.84 (18)	C9—C8—C13—C12	−0.9 (4)
N1—C14—C15—N5	18.8 (3)	C9—C10—C11—F1	−179.51 (18)
N2—C3—C4—O4	−176.77 (15)	C9—C10—C11—C12	−0.4 (3)
N2—C3—C4—C5	4.0 (2)	C10—C11—C12—C13	1.2 (4)
N3—C5—C6—O5	−178.36 (15)	C11—C12—C13—C8	−0.5 (4)
N3—C5—C6—N4	1.0 (2)	C13—C8—C9—C10	1.8 (3)
N4—C7—C8—C9	85.1 (2)	C14—N1—C1—C2	172.40 (17)
N4—C7—C8—C13	−94.3 (2)	C14—N1—C1—C18	51.1 (2)
N5—N6—C16—O1	0.5 (3)	C14—N1—C1—C19	−69.4 (2)
N5—N6—C16—C17	179.3 (3)	C15—O1—C16—N6	−0.3 (3)
N6—N5—C15—O1	0.2 (2)	C15—O1—C16—C17	−179.2 (2)
N6—N5—C15—C14	179.65 (19)	C15—N5—N6—C16	−0.4 (2)
C1—N1—C14—O2	−2.9 (3)	C16—O1—C15—N5	0.1 (2)
C1—N1—C14—C15	178.28 (17)	C16—O1—C15—C14	−179.43 (18)
C2—N2—C3—O3	177.59 (16)	C18—C1—C2—N2	65.4 (2)
C2—N2—C3—C4	−3.1 (2)	C18—C1—C2—N3	−115.50 (17)
C2—N3—C5—C4	0.1 (2)	C19—C1—C2—N2	−174.73 (15)
C2—N3—C5—C6	178.05 (15)	C19—C1—C2—N3	4.3 (2)
C3—N2—C2—N3	0.8 (2)	C20—N2—C2—N3	178.83 (16)
C3—N2—C2—C1	179.79 (15)	C20—N2—C2—C1	−2.2 (2)
C3—C4—C5—N3	−2.7 (3)	C20—N2—C3—O3	−0.6 (2)
C3—C4—C5—C6	179.50 (15)	C20—N2—C3—C4	178.63 (15)
C4—C5—C6—O5	−0.4 (2)		

### Hydrogen-bond geometry (Å, º)

**Table:**

*D*—H···*A*	*D*—H	H···*A*	*D*···*A*	*D*—H···*A*
O4—H4···O5	0.84	1.88	2.593 (2)	143
O1*W*—H1*WB*···O2	0.85	2.04	2.869 (2)	164
C20—H20*C*···N1	0.98	2.25	2.982 (3)	130
N1—H1···O5^i^	0.88	2.23	2.970 (2)	142
N4—H4*A*···O1*W*^ii^	0.88	2.48	3.074 (3)	126
C7—H7*A*···O1*W*^ii^	0.99	2.58	3.240 (3)	124
C10—H10···O5^iii^	0.95	2.50	3.393 (3)	158
C20—H20*A*···O4^iv^	0.98	2.46	3.394 (2)	160
C20—H20*B*···N6^v^	0.98	2.50	3.422 (3)	158
O1*W*—H1*WA*···O4^vi^	0.85	2.51	3.249 (3)	146
O1*W*—H1*WA*···O3^vi^	0.85	2.33	3.013 (3)	138

Symmetry codes: (i) −*x*+2, −*y*, −*z*+1; (ii) −*x*+2, −*y*+1, −*z*+1; (iii) −*x*+2, −*y*, −*z*+2; (iv) −*x*+1, −*y*, −*z*+1; (v) −*x*+1, −*y*, −*z*; (vi) *x*, *y*+1, *z*.
